# Supplementary material for: Ambient Air Quality and Emergency Hospital Admissions in Singapore: A Time-Series Analysis
Source: Int J Environ Res Public Health. 2022 Oct 16;19(20):13336. doi: 10.3390/ijerph192013336 (PMC9603816; doi:10.3390/ijerph192013336)

SUPPLEMENTARY INFORMATION

Table S1. Primary ED diagnosis in ICD-9 and ICD-10 codes used to categorize cardiovascular and respiratory diseases.

	ICD-9	ICD-10
Cardiovascular diseases	390–429, 439–59, 785	I00–I59, I70–I99, R00–R01, R03
Respiratory diseases	460–519, 786	J00–J99, R04–09

Table S2. Pearson correlation coefficient matrix between six air pollutants

	$PM_{2.5}$	PM_{10}	O_3	NO_2	SO_2	CO
$PM_{2.5}$		0.986287	0.305388	0.292939	0.189899	0.78873
PM_{10}			0.352508	0.260234	0.139743	0.784293
O_3				-0.16511	-0.15479	0.095209
NO_2					0.579546	0.4533
SO_2						0.133367
CO						

Figure S1. Association between daily average air pollutant levels and cumulative relative risk (lag 0-7 days) of emergency admissions with sensitivity analysis by varying the degrees of freedom. The solid red line represents the modelled cumulative risk. The shaded areas represent the 95% confidence intervals. The black solid horizontal line represents the reference value. The blue dashed vertical line indicates the air pollutant level recommended by WHO air quality guidelines.

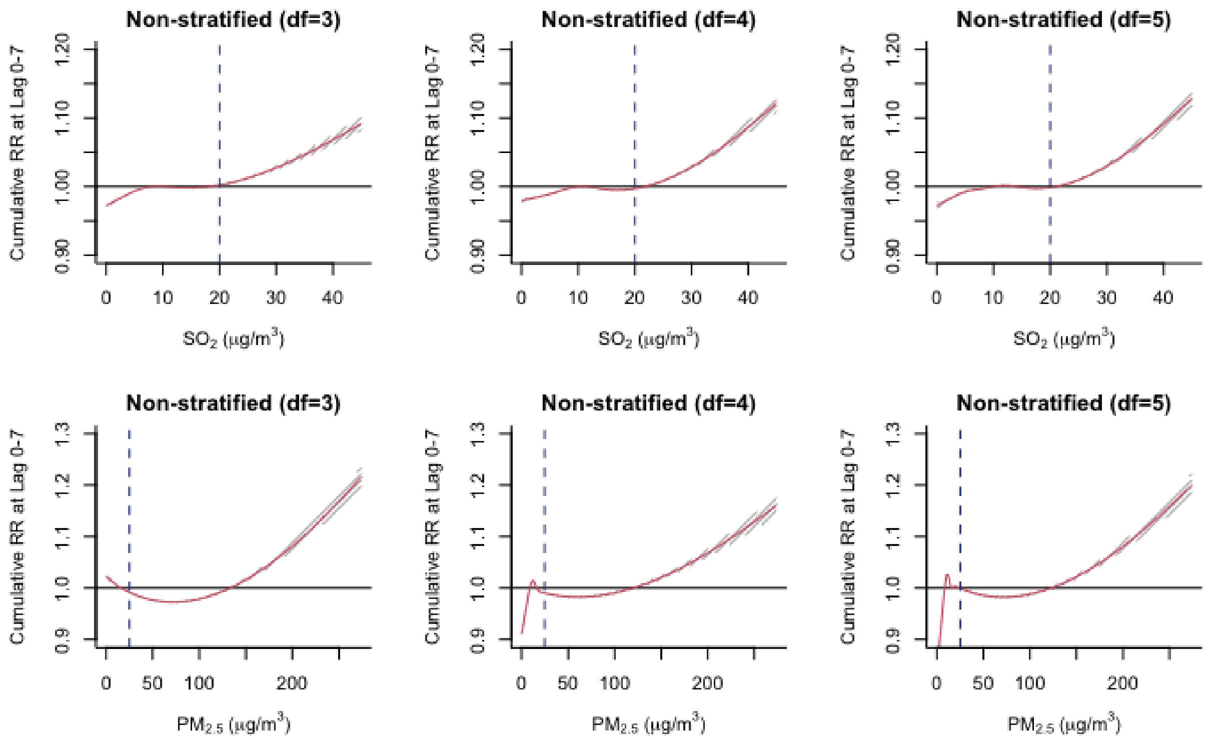

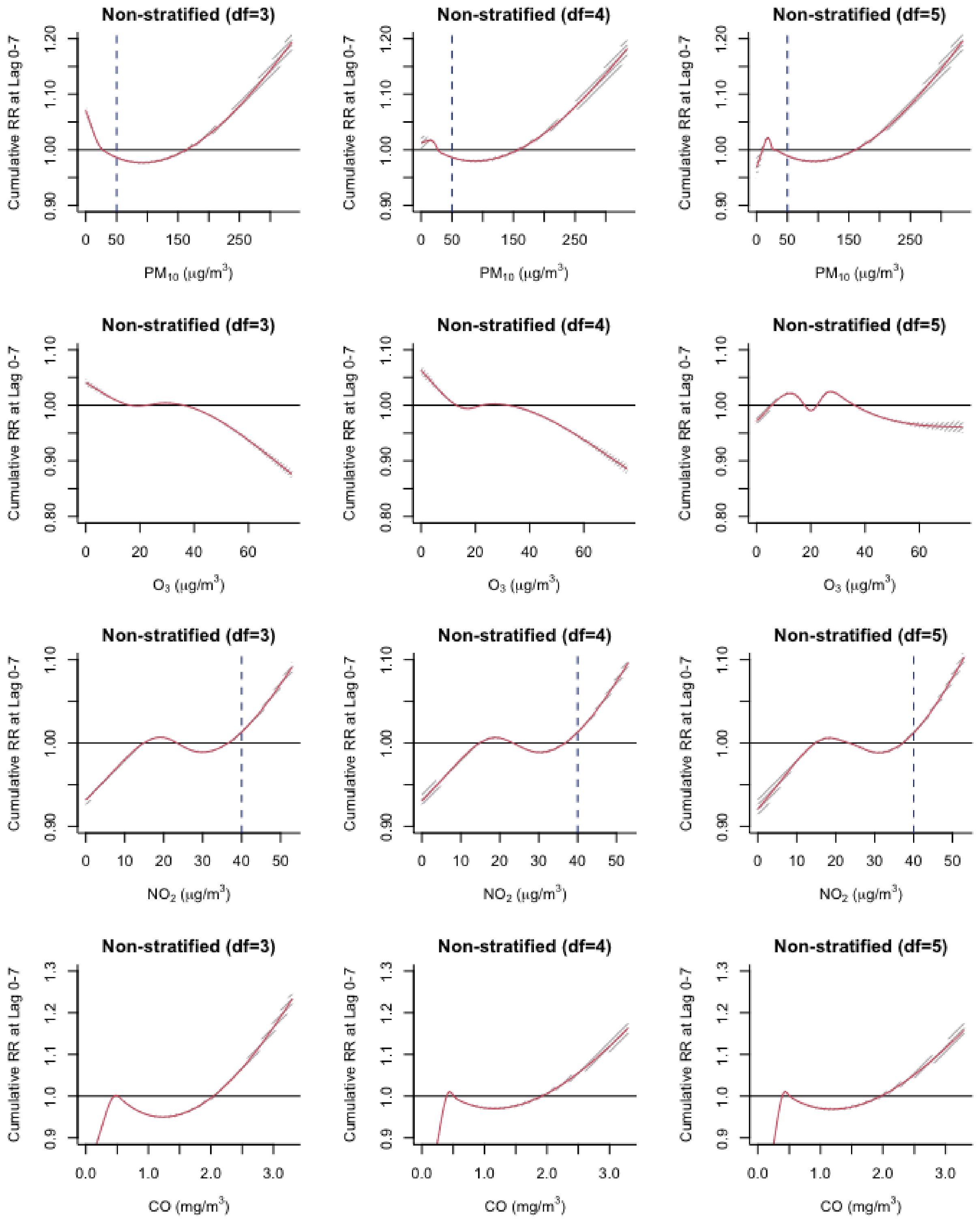

Non-stratified (df=3)
Non-stratified (df=4)
Non-stratified (df=5)
Cumulative RR at Lag 0-7
PM10 (μg/m3)
O3 (μg/m3)
NO2 (μg/m3)
CO (mg/m3)

Figure S2. Plot of fitted and actual daily emergency admission counts, using multipollutant model with $PM_{2.5}$

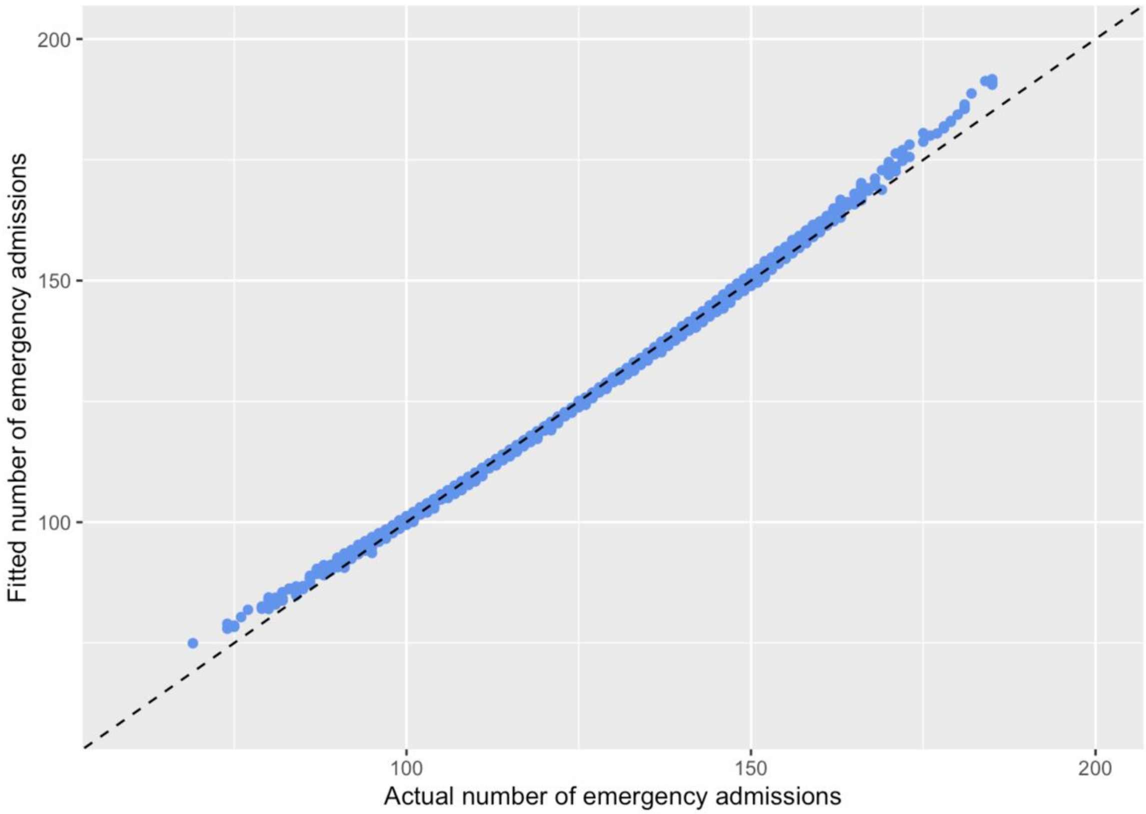

Figure S3. Association between daily average NO_2 and O_3 levels and cumulative relative risk (lag 0-7 days) of emergency admissions with sensitivity analysis by multipollutant models. The solid red line represents the modelled cumulative risk. The shaded areas represent the 95% confidence intervals. The black solid horizontal line represents the reference value. The blue dashed vertical line indicates the air pollutant level recommended by WHO air quality guidelines.

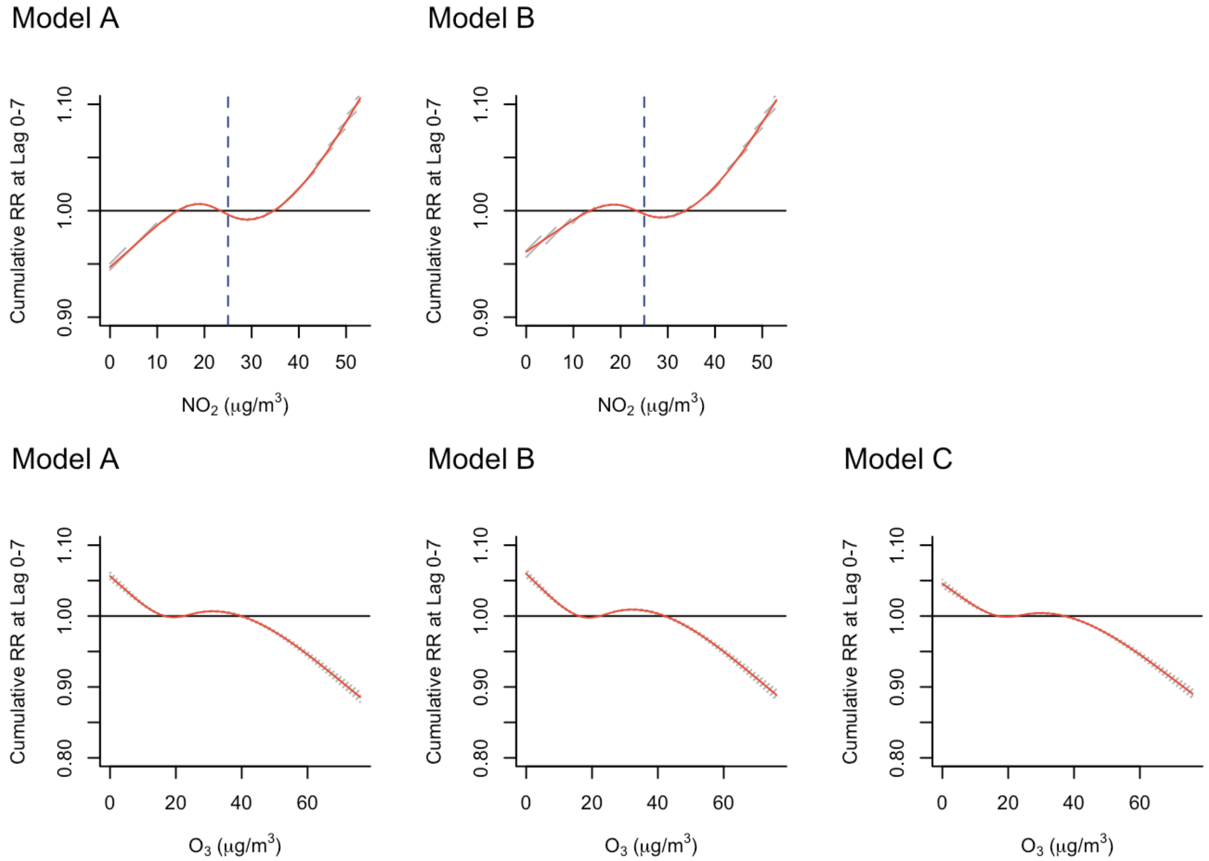

Supplement: Supplementary file 1 [file ijerph-19-13336-s001.zip › ijerph-1925856-supplementary.pdf]
